# Teriparatide (recombinant human parathyroid hormone 1-34) in postmenopausal women with osteoporosis: systematic review

**DOI:** 10.1590/S1516-31802008000500007

**Published:** 2008-09-04

**Authors:** Virgínia Fernandes Moça Trevisani, Rachel Riera, Aline Mizusaki Imoto, Humberto Saconato, Álvaro Nagib Atallah

**Keywords:** Osteoporosis, Fractures, bone, Postmenopause, Teriparatide, Review, Osteoporose, Fraturas ósseas, Pós-menopausa, Teriparatida, Revisão

## Abstract

**CONTEXT AND OBJECTIVE:**

Osteoporosis is defined as a disease characterized by low bone mass and deterioration of the bone tissue microarchitecture. Teriparatide stimulates the formation and action of osteoblasts, which are responsible for bone formation, thus promoting bone tissue increase. The aim was to assess the effectiveness and safety of teriparatide for treating postmenopausal osteoporosis.

**METHODS:**

A systematic review was conducted using the Cochrane Collaboration methodology.

**RESULTS:**

1) Teriparatide 20 µg or 40 µg versus placebo: there was a benefit from teriparatide, considering the following outcomes: reduction in the number of new vertebral and non-vertebral fractures, and increased whole-body, lumbar and femoral bone mineral density. 2) Teriparatide 40 µg versus alendronate 10 mg/day for 14 months: there was no statistical difference regarding the incidence of new vertebral or non-vertebral fractures, although in the group that received teriparatide there was greater bone mineral density increase in the whole body, lumbar column and femur. 3) Estrogen plus teriparatide 25 µg versus estrogen: there was a benefit, considering the following outcomes: reduction in the number of new vertebral fractures, and increased whole-body, lumbar and femoral bone mineral density after three years.

**CONCLUSION:**

When teriparatide is intermittently administered in low doses, it reduces the incidence of vertebral fractures (67%) and non-vertebral fractures (38%) and increases bone mineral density in the lumbar column and femur. There is a need for studies with longer observation in order to allow conclusions regarding the safety and duration of the therapeutic effects.

## RESUMO

**CONTEXTO E OBJETIVO:**

A osteoporose é definida como uma doença caracterizada por baixa massa óssea e deteriorização da microarquiquetura do tecido ósseo. O paratormônio estimula a formação e a ação dos osteoblastos responsáveis pela formação dos ossos, promovendo ganho de tecido ósseo. O objetivo foi determinar a efetividade e a segurança da teriparatida no tratamento da osteoporose pós-menopausa.

**MÉTODOS:**

Foi realizada uma revisão sistemática da literatura de acordo com a metodologia da Colaboração Cochrane.

**RESULTADOS:**

1) Teriparatida 20 µg ou 40 µg *versus* placebo: benefício da teriparatida considerando os desfechos redução do número de novas fraturas vertebrais e não-vertebrais, aumento da densidade mineral óssea corporal total, lombar e do fêmur. 2) Teriparatida 40 µg *versus* alendronato 10 mg/dia por 14 meses: não houve diferença estatística em relação a incidência de novas fraturas vertebrais ou não-vertebrais, porém, no grupo que recebeu a teriparatida, houve maior ganho de densidade mineral óssea corporal total, na coluna lombar e no fêmur (região intertrocantérica e triângulo de Wards). 3) Estrogênio mais teriparatida 25 µg *versus* estrogênio: houve benefício do estrogênio associado a teriparatida considerando os desfechos redução do número de novas fraturas vertebrais, aumento da densidade mineral óssea corporal total, lombar e do fêmur ao final dos três anos do estudo.

**CONCLUSÕES:**

A teriparatida, quando administrada em baixas doses e de forma intermitente, reduz as fraturas vertebrais (67%) e não vertebrais (38%) e aumenta a densidade mineral óssea na coluna lombar e no fêmur. Há necessidade de estudos de maior tempo de observação para permitir conclusões sobre a segurança e a duração dos efeitos terapêuticos.

## INTRODUCTION

Osteoporosis is a disease that is characterized by low bone mass and deterioration of the bone tissue microarchitecture, which cause bone fragility and increased risk of fractures.^1^ It is defined by bone densitometry as bone mineral density values lower than 2.5 standard deviations below the reference values.^2^

Fractures caused by low-level impact and increased mortality are the main outcomes from osteoporosis. The causes of the increased mortality are principally pulmonary thromboembolism, infections and systemic surgical and postoperative complications.^3,4^ The results from epidemiological studies show that there is a mortality rate of 14 to 34% over the first year following a hip fracture.^3,4^

A 50-year-old woman presents a risk of osteoporotic fracture during her remaining lifetime of 17.5% for the femoral neck, 15.6% for the vertebrae, 16% for the distal radius, and around 40% in any other location in the skeleton.^5^ The etiology of approximately 70% of hip fractures has been attributed to osteoporosis, with mortality ranging from 12 to 34%.^6^

Fractures most frequently occur in the vertebral column, hip or wrists, and they can occur even in the absence of falls. Between 50 and 100% of adults who have had one fracture will have a second one.^3,4,7^

Hip fractures may cause incapacity or death. Such patients present two to three times greater chance of dying than do individuals who have not had fractures.^8^ After suffering a hip fracture, only 30 to 40% of such individuals return to their usual activities.^8^

In the United States, approximately 10% of these patients become incapacitated following hip fractures, and 19% of them are institutionalized.^9^ In 2002, the expenditure on patients with osteoporosis exceeded 17 billion dollars, taking into account hospitalizations, home visits, physiotherapy and nursing.^10^

Prevention and treatment are undertaken through dietary counseling, exposure to the sun, exercises, prevention of falls, calcium and vitamin D supplementation and pharmacological treatment. Prevention begins in infancy, with a calcium-rich diet, exercise and exposure to the sun.^11-13^ Calcium supplementation (1000-1500 mg per day) and vitamin D supplementation reduce the risk of vertebral fracture, but not the risk of non-vertebral fracture. They increase the bone density in the lumbar column by 1.5 to 2%.^1,5,6^

The objectives of pharmacological treatment are prevention of fractures and post-fracture mortality, increased bone mineral density and reduced vertebral and non-vertebral pain following fractures.^1,5,6,11-13^ Bisphosphonates inhibit bone reabsorption by inhibiting osteoclasts. They increase bone mineral density by 10% and reduce the risk of vertebral and non-vertebral fracture by 40 to 50%.^1,5,6^ Calcitonin is an anti-reabsorption medication that reduces vertebral fracture by 20%, but not non-vertebral fracture, and it increases the bone mineral density in the vertebral column and femur.^1,5,6^ There is evidence that the use of calcitonin decreases the pain following vertebral fracture.^1,5,6^

Medication for selective estrogen receptor modulation (raloxifene) presents an estrogen-like effect on bones and fat metabolism and an antagonist effect on the breast and uterus. It leads to a slight increase in bone mineral density and a reduction in vertebral fractures, but not in non-vertebral fractures. Patients may present adverse effects consisting of thromboembolic events, with a relative risk of 3.29.^1,5,6^ Hormone replacement therapy increases the bone mineral density and reduces vertebral fractures by 34% and non-vertebral fractures by 27%, but has the adverse effects of increasing the risks of breast cancer and thromboembolic events. It must therefore not be indicated for treating osteoporosis.^1,5,6^

Teriparatide, the recombinant 1-34 fragment of human parathyroid hormone (rhPTH1-34) differs from the other therapeutic options for treating osteoporosis, such as bisphosphonates or calcitonin, which act by reducing bone reabsorption. Instead, it stimulates the formation and action of osteoblasts, which are the cells responsible for bone formation, thereby promoting increases in bone tissue.^14^

When teriparatide is intermittently administered in low doses, it presents an anabolic effect on bones, because it stimulates osteoblasts. This medication has been contraindicated for patients who are at risk of developing osteosarcoma, such as those with unexplained high alkaline phosphatase levels, those with Paget's disease, or those who have undergone irradiation on the bones. Teriparatide has also been contraindicated for patients with hyperparathyroidism or hypercalcemia.^1,5,6^

## OBJECTIVE

The aim was to determine the effectiveness and the safety of teriparatide for treating postmenopausal osteoporosis.

## MATERIALS AND METHODS

This systematic review of the literature was developed in accordance with the methodology of the Cochrane Collaboration and conducted at the Brazilian Cochrane Center, in the Universidade Federal de São Paulo — Escola Paulista de Medicina (Unifesp-EPM), on request from the Brazilian Ministry of Health.

The review only included randomized controlled clinical trials that evaluated the use of teriparatide for treating women with a diagnosis of natural or surgical postmenopausal osteoporosis. All clinical trials comparing teriparatide against placebo, hormone replacement, bisphosphonates, calcitonin or calcium in association with vitamin D could be included.

The outcomes evaluated were: a) percentage changes in bone mineral density, as quantified by dual-energy X-ray densitometry apparatus or by quantitative computed tomography of the femoral neck, femoral trochanter, hip, lumbar column or forearm; b) number of vertebral and non-vertebral fractures; c) toxicity, as measured by hypercalcemia, gastrointestinal symptoms or abandonment of treatment due to adverse effects.

### Search strategy for identifying studies

The search strategy included the following databases: Medical Literature Analysis and Retrieval System Online (Medline) (1996 to January 2005), Cochrane Systematic Review Database, Literatura Latino-Americana e do Caribe em Ciências da Saúde (Lilacs) (1996 to January 2005), Excerpta Medica Database (Embase) (1996 to January 2005) and congress abstracts within the fields of interest (rheumatology, orthopedics, osteoporosis and bones, from 1990 to December 2005). Pharmaceutical industry representatives were contacted to obtain access to unpublished data.

**Figure 1.**
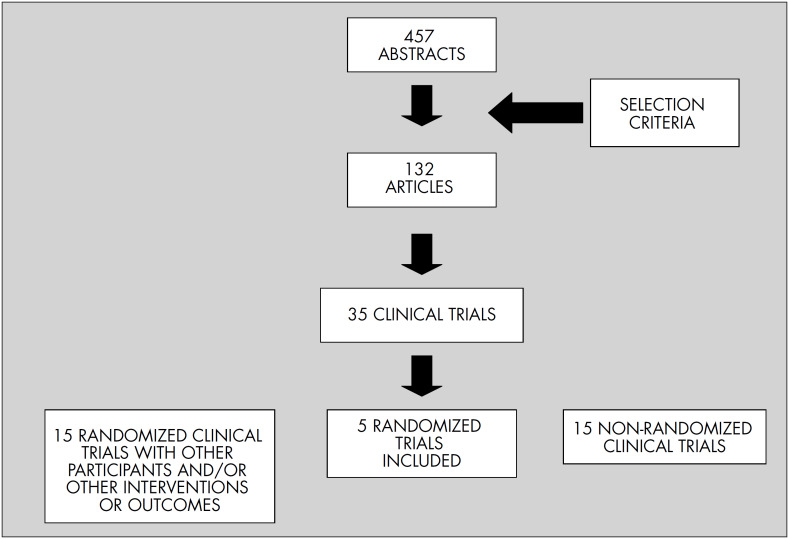
Result from the systematic review

The terms used in the databases were: osteoporosis, postmenopausal, parathyroid hormone (PTH) and teriparatide (Chart 1). Clinical trials were selected if they fulfilled the criteria that they were randomized controlled trials on postmenopausal populations and that they evaluated bone mineral density and fractures (Figure 1).

**Chart 1.** Search strategy

1. exp OSTEOPOROSIS/
2. osteoporos#s.tw.
3. bone loss$.tw.
4. or/1-3
5. Postmenopause/
6. (post menopaus$ or postmenopaus$ or post-menopaus$).tw.
7. 5 or 6
8. 4 and 7
9. exp Parathyroid Hormones/
10. parathyroid hormone$.tw.
11. Teriparatide.tw,rn.
12. (parathyrin or parathormone).tw.
13. (hpth or bpth).tw.
14. or/9-13
15. 8 and 14
16. clinical trial.pt.
17. randomized controlled trial.pt.
18. tu.fs.
19. dt.fs.
20. random$.tw.
21. (double adj blind$).tw.
22. placebo$.tw.
23. or/16-22
24. 15 and 23 meta-analysis
25. systematic review
26. overview

### Data extraction and methodological quality assessment

The search strategy identified the relevant articles. Each of these articles was reviewed by two independent reviewers. All the data were extracted by these two reviewers. Details relating to the population, treatment periods and demographic baseline were extracted independently. A third reviewer was consulted to help in resolving differences. The quality of each trial was evaluated independently by the two reviewers, using the validated quality assessment tool that was published by Jadad et al.^15^ in 1996.

### Statistical analysis and presentation of the results

The statistical analysis was carried out using the Manager program (RevMan, 2000), and in accordance with the Cochrane Collaboration Handbook.^16^ For dichotomous variables, the odds ratio (OR) method was used, with 95% confidence intervals (random effect model). When there was a statistical difference, the number needed to treat (NNT) or the number needed to harm (NNH) was calculated. For continuous variables, the weighted mean difference was calculated (random effect model) with the corresponding 95% confidence interval. If necessary, the original data were transformed into a logarithmic basis to obtain better distribution, or into scales that presented similar properties (the data on this scale would be the input for meta-analysis). In addition, if necessary, the continuous variables were subdivided for dichotomous analysis.

To analyze the sensitivity, the following strategy using the Review Manager (RevMan, 2000) was proposed:^16^

- reanalysis of the data using reasonable variation of values for lost data: when dichotomous variables were extracted, it was assumed that participants lost from the experimental group presented unsuccessful treatment and that losses from the control group presented improvement;
- reanalysis of the data using reasonable variation of the results from the studies, when there was some uncertainty in the results;
- reanalysis of the data using different statistical methods;
- statistical heterogeneity: it was planned that this would be evaluated in the studies by inspection of the graphical presentation (a dispersion graph in which the study weight or sample size was put on the y-axis, versus the risk ratio on the x-axis), and by the heterogeneity test (chi-squared test with n degrees of freedom, in which n was the number of studies that contributed data, minus one).

The studies included in the review are summarized in Table 1 and the studies excluded, together with the reason for exclusion, are identified in Table 2.^17-31^

**Table 1.** Summary of the clinical trials included

Author	Participants	Intervention
Neer et al.^17^*	1637	Teriparatide 20 µg or 40 µg SC/day versus placebo with 24 months of follow-up
Marcus et al.^18^*	1637	Teriparatide 20 µg or 40 µg SC/day versus placebo with duration of 24 months
Body et al.^19^	146	Teriparatide 40 µg SC + placebo versus oral alendronate 10 mg + placebo, with six months of follow-up
Lindsay et al.^20^	34	Teriparatide 400 U or 25 µg/day plus hormone replacement versus placebo + hormone replacement, with 36 months of follow-up
Finkelstein et al.^21^	50	Teriparatide 500 U or 40 µg + nafarelin acetate versus placebo + nafarelin acetate, with six months of follow-up

* Neer^17^ and Marcus^18^ published the same clinical trial, assessing different outcomes; PTH = parathyroid hormone; SC = subcutaneously.

**Table 2.** Summary of the clinical trials excluded

Study	Reason for exclusion
Buxton EC, Yao W, Lane NE. Changes in serum receptor activator of nuclear factor-kappaB ligand, osteoprotegerin, and interleukin-6 levels in patients with glucocorticoid-induced osteoporosis treated with human parathyroid hormone (1-34). J Clin Endocrinol Metab. 2004;89(7):3332-6.^17^	Participant group was different
Adis International Ltd. ALX 111: ALX1-11, parathyroid hormone (1-84) - NPS Allelix, PREOS, PTH, recombinant human parathyroid hormone, rhPTH (1-84). Drugs R D. 2003;4(4):231-5.^18^	Different intervention
Rehman Q, Lang TF, Arnaud CD, Modin GW, Lane NE. Daily treatment with parathyroid hormone is associated with an increase in vertebral cross-sectional area in postmenopausal women with glucocorticoid-induced osteoporosis. Osteoporos Int. 2003;14(1):77-81.^19^	Different outcome
Lane NE, Sanchez S, Modin GW, Genant HK, Pierini E, Arnaud CD. Bone mass continues to increase at the hip after parathyroid hormone treatment is discontinued in glucocorticoid-induced osteoporosis: results of a randomized controlled clinical trial. J Bone Miner Res. 2000;15(5):944-51.^20^	Participant group was different
Lane NE, Sanchez S, Modin GW, Genant HK, Pierini E, Arnaud CD. Parathyroid hormone treatment can reverse corticosteroid-induced osteoporosis. Results of a randomized controlled clinical trial. J Clin Invest. 1998;102(8):1627-33.^21^	Participant group was different
Cosman F, Nieves J, Woelfert L, Gordon S, Shen V, Lindsay R. Parathyroid responsivity in postmenopausal women with osteoporosis during treatment with parathyroid hormone. J Clin Endocrinol Metab. 1998;83(3):788-90.^22^	Different outcome
Hodsman AB, Fraher LJ. Biochemical responses to sequential human parathyroid hormone (1-38) and calcitonin in osteoporotic patients. Bone Miner. 1990;9(2):137-52.^23^	Different outcome
Kurland ES, Cosman F, McMahon DJ, Rosen CJ, Lindsay R, Bilezikian JP. Parathyroid hormone as a therapy for idiopathic osteoporosis in men: effects on bone mineral density and bone markers. J Clin Endocrinol Metab. 2000;85(9):3069-76.^24^	Participant group was different
Kaufman JM, Orwoll E, Goemaere S, et al. Teriparatide effects on vertebral fractures and bone mineral density in men with osteoporosis: treatment and discontinuation of therapy. Osteoporos Int. 2005;16(5):510-6.^25^	Participant group was different
Orwoll ES, Scheele WH, Paul S, et al. The effect of teriparatide [human parathyroid hormone (1-34)] therapy on bone density in men with osteoporosis. J Bone Miner Res. 2003;18(1):9-17.^26^	Participant group was different
Jiang Y, Zhao JJ, Mitlak BH, Wang O, Genant HK, Eriksen EF. Recombinant human parathyroid hormone (1-34) [teriparatide] improves both cortical and cancellous bone structure. J Bone Miner Res. 2003;18(11):1932-41.^27^	Different outcome
Hodsman AB, Kisiel M, Adachi JD, Fraher LJ, Watson PH. Histomorphometric evidence for increased bone turnover without change in cortical thickness or porosity after 2 years of cyclical hPTH(1-34) therapy in women with severe osteoporosis. Bone. 2000;27(2):311-8.^28^	Different outcome
Hodsman AB, Hanley DA, Ettinger MP, et al. 3Efficacy and safety of human parathyroid hormone-(1-84) in increasing bone mineral density in postmenopausal osteoporosis. J Clin Endocrinol Metab. 2003;88(11):5212-20.^29^	Hormone other than parathyroid (1-84)
Black DM, Greenspan SL, Ensrud KE, et al. The effects of parathyroid hormone and alendronate alone or in combination in postmenopausal osteoporosis. N Engl J Med. 2003;349(13):1207-15.^30^	Hormone other than parathyroid (1-84)
Hodsman AB, Fraher LJ, Watson PH, et al. A randomized controlled trial to compare the efficacy of cyclical parathyroid hormone versus cyclical parathyroid hormone and sequential calcitonin to improve bone mass in postmenopausal women with osteoporosis. J Clin Endocrinol Metab. 1997;82(2):620-8.^31^	Different intervention

The studies included were:

-**Neer et al.**^32^ ***Effect of parathyroid hormone (1-34) on fracture and bone mineral density in postmenopausal women with osteoporosis***. This was a paper originating from a double-blind randomized clinical trial with 24 months of follow-up. It included 1637 women who had been postmenopausal for at least five years and who had suffered at least one moderate or two slight non-traumatic fractures. An additional inclusion criterion for women who had had fewer than two fractures was presentation of hip or lumbar column bone mineral density of at least one standard deviation below the mean. They were administered teriparatide 20 µg or 40 µg daily, or placebo. The outcomes were: 1) new fractures; 2) bone mineral density of the lumbar column, proximal femur, radius and whole body measured on the Lunar, Hologic or Norland densitometry apparatus; and 3) adverse effects. The loss during follow-up was 311 patients. This study was performed in the United States with pharmaceutical industry sponsorship.

-**Marcus et al.**^33^ ***The skeletal response to teriparatide is largely independent of age initial bone mineral density, and prevalent vertebral fracture in postmenopausal women with osteoporosis. Effect of parathyroid hormone (1-34) on fracture and bone mineral density in postmenopausal women with osteoporosis***. This paper originated from the same clinical trial as described in the preceding paper, but assessing different outcomes, since the patients included were subdivided into three groups according to age group (less than 65 years old, from 65 to 75 years old, and more than 75 years old).

-**Body et al.**^34^ ***A randomized double-blind trial to compare the efficacy of teriparatide [recombinant human parathyroid hormone (1-34)] with alendronate in postmenopausal women with osteoporosis***. This was a randomized clinical trial with six months of follow-up. It included 146 women who had been postmenopausal for at least five years and were between 35 and 85 years old. Their bone mineral density in the hip and vertebral column was lower than 2.5 standard deviations below the mean, as evaluated using the Lunar or Hologic densitometry apparatus. The baseline characteristics of the participants appeared to be similar. The exclusion criteria were the presence of other bone metabolism diseases, nephrolithiasis, malabsorption, kidney or liver failure, use of medications that affect bone metabolism and use of alcoholic drinks. The interventions were parathyroid hormone 40 µg plus placebo *versus* alendronate 10 mg/day plus placebo. The outcomes were: 1) bone mineral density evaluated by tomography; 2) new fractures; 3) biochemical markers; and 4) adverse effects. The loss during follow-up was three patients. This study was performed in the United States, and there was no information regarding sponsorship.

-**Lindsay et al.** ^35^ ***Randomized controlled study of effect of parathyroid hormone on vertebral-bone mass and fracture incidence among postmenopausal women on estrogen with osteoporosis***. This was a randomized clinical trial with 36 months of follow-up. It included 34 women who were undergoing hormone replacement. Postmenopausal osteoporosis was defined as the presence of at least one non-traumatic fracture or bone mineral density lower than 2.5 standard deviations below the mean. The participants' baseline characteristics appeared to be similar. The interventions were: teriparatide 400 U or 25 µg daily plus hormone replacement, or hormone replacement alone. The outcomes were: 1) evaluation of bone mineral density in the lumbar column; 2) evaluation of bone mineral density in other locations; and 3) new fractures. The loss during follow-up was four participants. This study was performed in the United States, with sponsorship from Rhône-Poulenc Rorer, who supplied the teriparatide.

-**Finkelstein et al.**^36^ ***Parathyroid hormone for the prevention of bone loss induced by estrogen deficiency***. This was a randomized clinical trial with six months of follow-up. It included 50 women between 20 and 44 years old who had a history of endometriosis. The participants' baseline characteristics appeared to be similar. The exclusion criteria were hyperthyroidism, hyperparathyroidism, Cushing, hyperprolactinemia, anorexia nervosa, chronic liver disease, renal insufficiency, alcoholism and use of corticoids. The interventions were parathyroid hormone 500 U or 40 µg plus nafarelin acetate, or nafarelin acetate alone. The outcomes were: 1) bone mineral density; and 2) adverse effects. The loss during follow-up was ten participants. This study was performed in the United States, with sponsorship from the pharmaceutical industry.

## RESULTS

For the purposes of statistical analysis, the comparisons were made according to the control group: 1) placebo; 2) alendronate; 3) hormone replacement; and 4) other interventions.

### 1. Teriparatide versus placebo

This megatrial,^32,33^ which included 1637 postmenopausal women, gave rise to two papers (Neer et al.,^32^ and Marcus et al.,^33^) that assessed different outcomes and have therefore been included separately in this review.

There was a statistically significant benefit from using teriparatide 20 µg or 40 µg, in relation to placebo, considering the following outcomes: reduction in the number of new vertebral fractures (relative risk, RR 0.35; confidence interval, CI 0.22 to 0.55, for 20 µg; and RR 0.29; CI 0.18 to 0.48, for 40 µg); reduction in the number of new non-vertebral fractures (RR 0.54; CI 0.37 to 0.79, for 20 µg; and RR 0.5; CI 0.34 to 0.74, for 40 µg); increased whole-body bone mineral density (RR 3.1; CI 1.65 to 4.55, for 20 µg; and RR 4.5; CI 2.78 to 6.22, for 40 µg); increased lumbar bone mineral density (RR 9.6; CI 7.79 to 9.41, for 20 µg; and RR 12.6; CI 11.62 to 13.58, for 40 µg); and increased femoral bone mineral density (RR 3.6; CI 2.75 to 4.45, for 20 µg; and RR 4.6; CI 3.71 to 5.49, for 40 µg). There was no difference between the two doses of teriparatide when considering whole-body bone mineral density (RR -11.4; CI -3.08 to 0.28), lumbar bone mineral density (RR -4.00; CI -5.07 to -2.93) or femoral bone mineral density (RR -1.00; CI -0.95 to -0.05).

With regard to abandonment of treatment, there was no statistical difference between teriparatide 20 µg and placebo (p = 0.52 overall and p = 0.32 with heterogeneity correction). However, there was statistically greater abandonment in the teriparatide 40 µg group than for placebo (p < 0.01). Comparing the two doses of teriparatide, there was also statistically greater abandonment in the 40 µg group (p < 0.01).

The following side effects were more frequent in the teriparatide group, independent of the dose, than in the placebo group: headache (RR 1.4; CI 1.03 to 1.91), nausea (RR 2.34; CI 1.38 to 3.25), cramps (RR 3.22; CI 1.19 to 8.72), hypercalcemia (RR 9.73; CI 5.35 to 17.67) and formation of anti-PTH antibodies (RR 17.42; CI 12.39 to 126.81). There was no statistically significant difference regarding occurrences of dizziness (RR 1.49; CI 0.98 to 2.28).

The study by Marcus et al.^33^ demonstrated that the reductions in the risk of new fractures in the teriparatide group were similar, independent of age (p = 0.558). Likewise, the increase in bone mineral density did not show dependency on the baseline bone mineral density (p = 0.615). The relative risks of new fractures were similar between the subgroups and there was no association with the baseline bone mineral density (p = 0.817). Furthermore, there was no statistically significant difference between the two doses of teriparatide, in relation to reduction in the risk of new fractures (p = 0.649).

### 2. Teriparatide versus alendronate

There was no statistical difference in relation to the incidence of new vertebral or non-vertebral fractures (RR 0.3; CI 0.09 to 1.05).^34^ However, in the group that received teriparatide, there were greater increases in bone mineral density in the whole body, lumbar column and femur (intertrochanteric and Ward's triangle regions). There was no bone mass increase in the distal radius of either group. The following adverse effects were more frequent in the teriparatide group: cramps (RR 13.00; CI 0.75 to 226.62); elevated serum calcium on at least one occasion (RR 14.04; CI 13.97 to 49.59); elevation of serum calcium on at least two occasions (RR 18.74; CI 1.11 to 316.15); and elevation of urinary calcium (RR 9.66; CI 0.57 to 163.19). The worsening of lumbar pain was greater in the alendronate group (RR 0.29; CI 0.10 to 0.83).

### 3. Teriparatide plus estrogen versus estrogen

There was a statistically significant benefit from using estrogen in association with teriparatide, considering the following outcome:^35^ reduction in the number of new vertebral fractures (RR 0.15; CI 0.03 to 0.73). The data relating to bone mineral density could not be extracted for analysis but, by the end of the three years of the study, there had been increases of 13% in lumbar bone mineral density, 2.7% in femoral bone mineral density and 7.8% in whole-body bone mineral density in the teriparatide/estrogen group. There was practically no increase in the group that received estrogen alone.

### 4. Others: teriparatide plus nafarelin versus nafarelin

The study that compared teriparatide with placebo (nafarelin versus nafarelin plus teriparatide 40 µg) for preventing bone mass loss among women with medication-induced menopause (nafarelin gonadotrophic hormone)^36^ showed that, after six months of treatment, there was a bone mass increase of 3.4% in the teriparatide group and a bone mass loss of 3.5% in the placebo group, considering the lumbar column and whole body. However, there was a slight reduction in the bone mineral density of the femoral neck in both groups.

## DISCUSSION

The results from this systematic review have shown that the use of teriparatide reduced the number of vertebral fractures (67%) and non-vertebral fractures (38%) and increased the bone mineral density of the lumbar column and femur, over the period when it was used. However, it is still not possible to assess the duration of these beneficial effects after the treatment was stopped.

The adverse effects attributed to teriparatide did not stop the patients from continuing with the treatment. However, some important points must be made with regard to the use and safety of this medication. Previous studies have demonstrated increased incidence of osteosarcoma in rodents treated with PTH, while there have not been any reports of cases in monkeys.^6^ The studies among humans have not had sufficient duration to find definitive evidence in this respect. Therefore, this medication must still be contraindicated for patients who are at risk of developing osteosarcoma, such as those with unexplained high alkaline phosphatase levels, those with Paget's disease, or those who have undergone irradiation on the bones. Moreover, it must be contraindicated for patients with hyperparathyroidism or hypercalcemia.

With regard to the ideal dose for teriparatide, the study by Neer et al., which was initially envisaged to last for three years but was halted after 19 months of use of the medication, showed that there were no differences between the therapeutic effects from 20 µg and 40 µg.^32^ Thus, a dose of 20 µg for a maximum period of 24 months was established.^17^

Because of its high cost (approximately 14,000 dollars for two years^1,5,6^) and considering that there are other effective interventions that are economically more viable for treating osteoporosis, teriparatide must only be prescribed after the other therapeutic options have been exhausted.

## CONCLUSIONS

Teriparatide administered intermittently at doses of 20 µg or 40 µg, in comparison with placebo, reduced the incidence of new vertebral and non-vertebral fractures and improved the bone mineral density of the whole body and lumbar column, without apparent correlation with any serious adverse effects. Teriparatide (40 µg) was superior to alendronate (10 mg/day), considering bone mass increases for the whole body, lumbar column and femur (intertrochanteric and Ward's triangle regions), but there was no difference regarding the incidence of new fractures.
